# Phenformin inhibits growth and epithelial-mesenchymal transition of ErbB2-overexpressing breast cancer cells through targeting the IGF1R pathway

**DOI:** 10.18632/oncotarget.19466

**Published:** 2017-07-22

**Authors:** Zhiying Guo, Ming Zhao, Erin W. Howard, Qingxia Zhao, Amanda B. Parris, Zhikun Ma, Xiaohe Yang

**Affiliations:** ^1^ Julius L. Chambers Biomedical/Biotechnology Research Institute, Department of Biological and Biomedical Sciences, North Carolina Central University, Kannapolis, North Carolina, USA

**Keywords:** phenformin, breast cancer, epithelial-mesenchymal transition, insulin-like growth factor 1 receptor, MMTV-ErbB2 transgenic mice

## Abstract

Reports suggest that metformin, a popular anti-diabetes drug, prevents breast cancer through various systemic effects, including insulin-like growth factor receptor (IGFR) regulation. Although the anti-cancer properties of metformin have been well-studied, reports on a more bioavailable/potent biguanide, phenformin, remain sparse. Phenformin exerts similar functional activity to metformin and has been reported to impede mammary carcinogenesis in rats. Since the effects of phenformin on specific breast cancer subtypes have not been fully explored, we used ErbB2-overexpressing breast cancer cell and animal models to test the anti-cancer potential of phenformin. We report that phenformin (25–75 μM) decreased cell proliferation and impaired cell cycle progression in SKBR3 and 78617 breast cancer cells. Reduced tumor size after phenformin treatment (30 mg/kg/day) was demonstrated in an MMTV-ErbB2 transgenic mouse syngeneic tumor model. Phenformin also blocked epithelial-mesenchymal transition, decreased the invasive phenotype, and suppressed receptor tyrosine kinase signaling, including insulin receptor substrate 1 and IGF1R, in ErbB2-overexpressing breast cancer cells and mouse mammary tumor-derived tissues. Moreover, phenformin suppressed IGF1-stimulated proliferation, receptor tyrosine kinase signaling, and epithelial-mesenchymal transition markers *in vitro*. Together, our study implicates phenformin-mediated IGF1/IGF1R regulation as a potential anti-cancer mechanism and supports the development of phenformin and other biguanides as breast cancer therapeutics.

## INTRODUCTION

Recent advances in cancer research suggest a link between metformin, a commonly prescribed anti-type 2 diabetes drug, and reduced cancer risk, including prostate, lung, pancreatic, and breast cancer [1–3]. Results from animal models and cultured cell lines provide general support for the anti-cancer effects of metformin, despite inconsistent data in non-diabetic patients [4–6]. Metformin-mediated anti-cancer activities have been associated with both systemic and cellular regulatory activities, including lowering insulin levels, increasing insulin sensitivity, and activation of AMP-activated protein kinase (AMPK)/inhibition of mammalian target of rapamycin (mTOR) [3, 7]. It appears that metformin-induced insulin receptor (IR) and insulin growth factor (IGF) receptor (IGFR) inactivation stimulates the regulatory network as well [8, 9]. Increasing evidence supports metformin as a promising anti-cancer agent with benefits to patients with breast and other cancers. In order to further elucidate the anti-cancer potential of metformin, it is critical to investigate other biguanide drugs, such as phenformin.

While the anti-cancer mechanism of metformin has been well-studied, the anti-cancer effects of phenformin remain to be explored in depth. As biguanide analogs, phenformin has similar effects on diabetes and cancer as metformin, including the inhibition of mitochondrial complex I [3, 10, 11]; however, the hydrophobic moiety of phenformin increases its cellular uptake as compared to metformin. Indeed, Janzer *et al*. reported that phenformin has more biological activity than metformin in regards to the inhibition of mammary carcinogenesis—possibly due to increased bioavailability [12]. Although the anti-cancer potential of phenformin was initially explored decades ago, recent studies have confirmed that phenformin can inhibit mammary tumorigenesis with alterations in angiogenesis, proliferation, apoptosis, and epithelial-mesenchymal transition (EMT) in various cell and animal models of early and late stage breast cancers [13–16]. Nevertheless, the anti-cancer properties of phenformin have only come to light in the past decade and the mechanisms of phenformin that are specific to breast cancer prevention and treatment need to be explored. Recent findings necessitate further mechanistic investigation of the anti-cancer effects of phenformin in cellular and animal models of breast and other cancers.

As a focus of our current study, EMT is often implicated in breast cancer development and invasiveness, as well as tumor heterogeneity [17, 18]. EMT endows metastatic properties upon cancer cells to promote migration, invasion, and subsequent dissemination [19, 20]; thus, bolstering the importance of EMT inhibition in cancer therapy development. Pathways that regulate EMT, including tumor growth factor-β (TGF-β)/Smad, receptor tyrosine kinase (RTK), Wnt, and Notch signaling pathways [21–23], are anti-cancer drug targets. In particular, the RTKs involved in the EMT process consist of epidermal growth factor receptors (EGFRs) and IGFRs. Studies have determined that IGFs promote the proliferation, survival, metastatic ability, and EMT in breast cancer cells [23–26]. Since phenformin has previously been reported to alter the EMT phenotype and decrease the invasive capacity of breast cancer cells [16], the anti-cancer benefits of phenformin on EMT warrant further investigation that will provide essential mechanistic insight.

Our research focuses on novel cancer preventative and therapeutic strategies targeting the ErbB2-overexpressing/ErbB2-positive (ErbB2^+^) breast cancer subtype, which is associated with poor prognosis and therapeutic resistance [27, 28]. Importantly, the ErbB2^+^ breast cancer subtype has been reported to be more responsive than the ErbB2-negative subtype to metformin and other biguanides [29, 30]. Based on our previous work and the premise that the effects of phenformin on individual breast cancer subtypes have not been fully explored, it is intriguing to explore the effects of phenformin on ErbB2-overexpressing breast cancer models. To this end, our current study investigates the anti-tumor activity of phenformin on ErbB2-overxpressing breast cancer. We found that phenformin significantly inhibits proliferation, migration, invasion, and EMT in ErbB2-overexpressing breast cancer cells and blocks tumor growth in a syngeneic mammary tumor model, which were associated with the inhibition of IGF1R stimulation. All in all, our *in vitro* and *in vivo* data support phenformin as a promising candidate for ErbB2^+^ breast cancer treatment and provides the foundation for future studies on the anti-cancer mechanisms of biguanide drugs.

## RESULTS

### Phenformin inhibits the proliferation and clonogenic survival of ErbB2-overexpressing breast cancer cells *in vitro*

**Figure 1:**
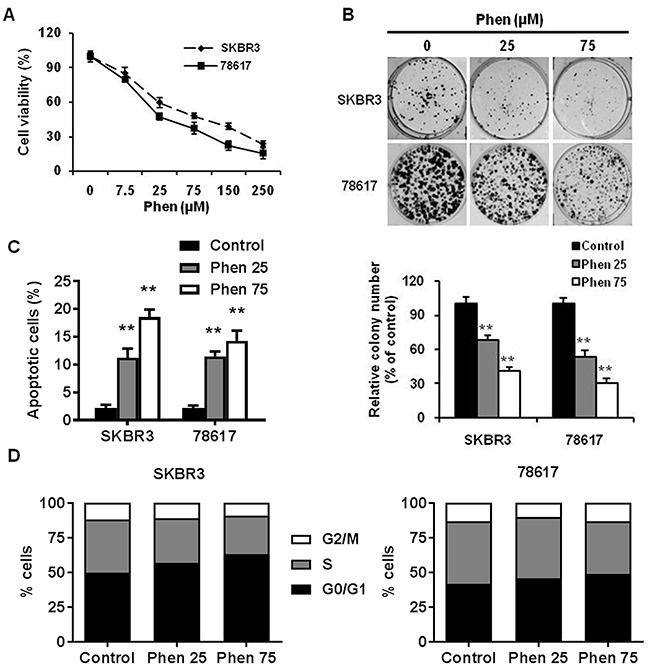
Phenformin inhibits the proliferation and clonogenic survival of ErbB2-overexpressing breast cancer cells *in vitro* **(A)** SKBR3 and 78617 cells were treated with phenformin (0, 7.5, 25, 75, 150, or 250 μM) for 5 days, followed by MTT assay to determine cell proliferation. **(B)** SKBR3 and 78617 cells were treated with phenformin (0, 25, or 75 μM) for 2 weeks. Then, to quantify the colony formation efficiency, the cells were stained with crystal violet. Representative images of the crystal violet-stained cells are shown in the top panel. In the lower panel, the graph displays the statistical analysis of colonies from each group in triplicate. Data are presented as the mean ± standard error of the mean (S.E.) of three independent samples (** p<0.01). **(C)** SKBR3 and 78617 cells were treated with phenformin (0, 25, or 75 μM) for 48 hours. To measure the percentage of apoptotic cells, Annexin V-FITC and PI staining was detected by flow cytometry. Data are presented as the mean ± S.E. (** p<0.01). **(D)** SKBR3 and 78617 cells were treated with phenformin (0, 25, or 75 μM) for 24 hours. Cell cycle distribution was measured by FACS using a PI staining assay. The values shown indicate the percentage of cells in G0/G1, S, and G2/M phase of the cell cycle.

To test the effects of phenformin on cell proliferation *in vitro*, we performed MTT assays on SKBR3 and 78617 cells. In Figure 1A, phenformin (25-250 μM) significantly inhibited cell proliferation dose-dependently in both cell lines. To support our MTT results, we further examined the effect of phenformin on colony formation potential using a clonogenic assay. Accordingly, phenformin (25 and 75 μM) inhibits the formation of colonies in a dose-dependent manner in both of the cell lines (Figure 1B). Moreover, phenformin (25 and 75 μM) for 48 hours induced apoptosis in SKBR3 and 78617 (Figure 1C, Supplementary Figure 1A). Cell cycle analysis of treated cells indicated that phenformin (25 and 75 μM) for 24 hours induced G0/G1 arrest, which was accompanied by a parallel decrease in the percentage of cells in S phase (Figure 1D, Supplementary Figure 1B). Together, these results indicate that phenformin has effective anti-proliferative activities, which are both cytostatic and cytotoxic, in ErbB2-overexpressing breast cell lines *in vitro*.

### Phenformin inhibits ErbB2-overxpressing mammary tumor development in the syngeneic graft mouse model

**Figure 2:**
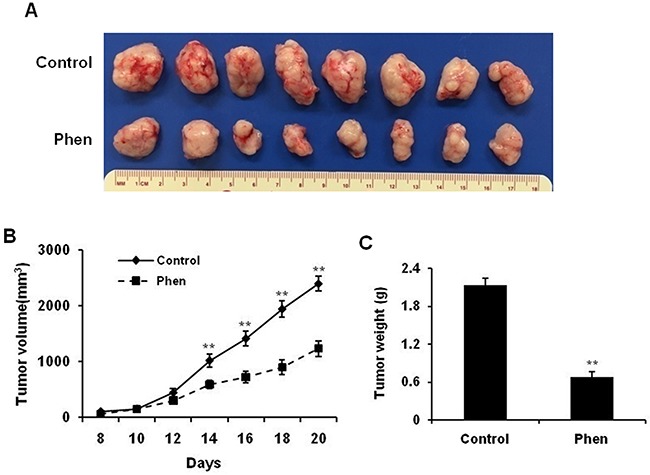
Phenformin inhibits ErbB2-overexpressing mammary tumor development in the syngeneic graft mouse model MMTV-ErbB2 tumor-derived 78617 cells were cultured with regular DMEM medium and then trypsinized. After adjusting cell number based on viability, 1×10^6^ viable 78617 cells were injected subcutaneously into the flank of MMTV-ErbB2 transgenic mice. Phenformin (30 mg/kg/day) or saline (control) was then intraperitoneally injected for 20 days. Tumor volumes were measured every other day from the 8^th^ day after injection until the 20^th^ day. **(A)** Representative images are shown of grafted tumors from control and phenformin-treated mice. Graphs of tumor growth curves (**B**) and tumor weight (**C**) are depicted. Data are presented as the mean ± S.E. (** p<0.01).

To investigate the potential anti-cancer activity of phenformin *in vivo*, we used a syngeneic graft model. To this end, MMTV-ErbB2 transgenic mice were inoculated with 78617 cells, which were derived from mammary tumors of MMTV-ErbB2 mice [31]. As shown in Figure 2A and 2B, phenformin treatment (30 mg/kg/day) significantly inhibited tumor growth by day 20 as compared to the saline-treated mice. Consistently, the average tumor weight at the end of the experiment was substantially lower in the phenformin-treated group than in the control group (Figure 2C). These results corroborate our *in vitro* data and indicate that phenformin inhibits tumor growth in our mouse model of breast cancer.

### Phenformin inhibits cell migration and invasion in ErbB2-overexpressing breast cancer cells

**Figure 3:**
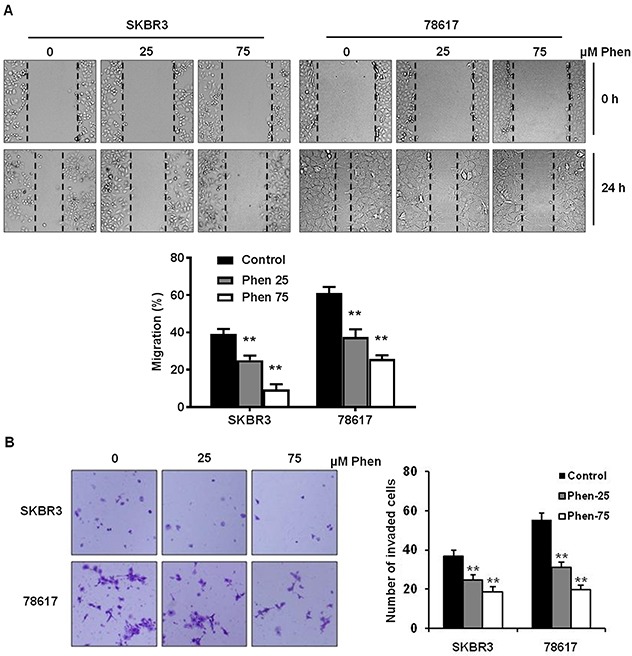
Phenformin inhibits cell migration and invasion in ErbB2-overexpressing breast cancer cells **(A)** The migration of cells treated with phenformin (0, 25, or 75 μM) for 24 hours was determined by a wound healing assay. The upper panel shows SKBR3 and 78617 cells at 0 hours and 24 hours after the initial wound was formed. Representative images were captured at 100× magnification and the dashed lines indicate the boundaries of the wound. The lower panel depicts the percent of the wound width that the cells migrated after 24 hours. Data are presented as the mean ± S.E. (** p<0.01). **(B)** The cell invasion capacity of SKBR3 and 78617 cells treated with phenformin (0, 25, or 75 μM) for 24 hours was measured by matrigel invasion assays. Representative images of crystal violet-stained cells are shown at 24 hours. The graph in the panel to the right shows the number of cells that invaded the lower chamber. Data are shown as the mean ± S.E. (** p<0.01).

Cell motility is associated with aggressive breast cancer phenotypes; therefore, we investigated the effect of phenformin on cell migration and invasion using wound healing and invasion chamber assays, respectively, in SKBR3 and 78617 cells. As shown in Figure 3A, phenformin (25 and 75 μM) significantly inhibited cell migration in both cell lines. Importantly, using mitomycin C to control for cell proliferation, we determined that phenformin-induced inhibition of migration was not the result of defective cell proliferation (Supplementary Figure 2A). We also observed that phenformin induced an epithelial-like morphological phenotype, particularly in the 78617 cells (Supplementary Figure 2B). Moreover, phenformin (25 and 75 μM) markedly reduced cell invasion, as indicated by a decreased number of cells that transmigrated through the matrigel inserts upon phenformin treatment in the invasion assay (Figure 3B). Similar results from a Boyden chamber assay in the absence of matrigel were also observed (Supplementary Figure 2C). Our data reveal that phenformin treatment significantly attenuates cell migration and invasion in breast cancer cell lines.

### Phenformin inhibits EMT in ErbB2-overexpressing breast cancer cells

**Figure 4:**
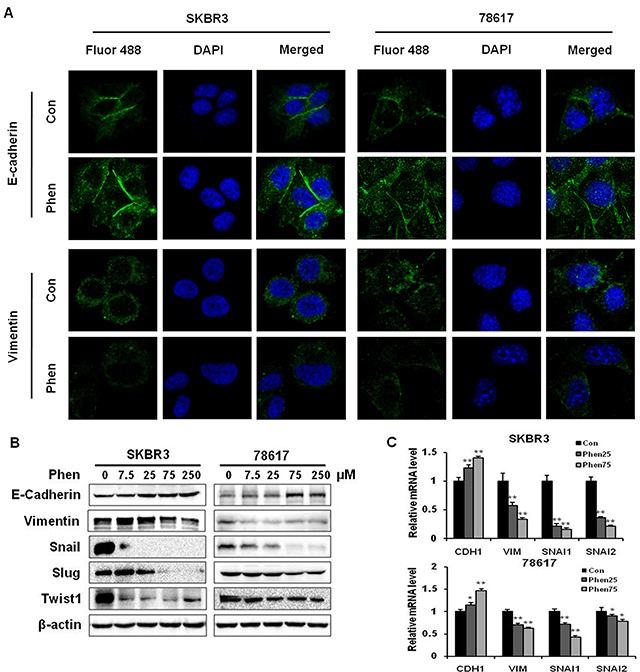
Phenformin inhibits EMT in ErbB2-overexpressing breast cancer cells *in vitro* **(A)** An immunofluorescence assay was used to determine EMT marker (E-cadherin and vimentin) expression in SKBR3 and 78617 cells treated with phenformin (0 or 75 μM) for 24 hours. Nuclei were stained with DAPI (blue), while E-cadherin and vimentin are indicated by Fluor 488 green fluorescence in the indicated panels. **(B)** Western blot analysis of EMT markers in SKBR3 and 78617 cells treated with phenformin (0, 7.5, 25, 75, or 250 μM) for 72 hours is shown. **(C)** qPCR was performed to quantify *CDH1* (E-cadherin), *VIM* (vimentin), *SNAI1* (Snail), and *SNAI2* (Slug) mRNA transcript levels in SKBR3 and 78617 cells after treatment with phenformin (0, 25, or 75 μM) for 24 hours. *GAPDH* and *actb* were used as controls in SKBR3 and 78617 cells, respectively. Data are normalized to the control sample for each gene and represent the mean ± S.E. of triplicate samples (* p<0.05; ** p<0.01).

In order to investigate whether phenformin decreases breast cancer cell invasion by inhibiting EMT, we analyzed several EMT markers in SKBR3 and 78617 cells. As shown in Figure 4A, immunofluorescence results showed that phenformin (75 μM) noticeably increased protein levels of E-cadherin, an epithelial marker, and decreased protein levels of vimentin, a mesenchymal marker, in both cell lines. Consistently, Western blot analysis demonstrated that phenformin (7.5 – 250 μM) strikingly increased the expression of E-cadherin, while decreasing the levels of vimentin and other mesenchymal markers. Among the EMT markers, phenformin remarkably downregulated Snail, Slug, and Twist1, especially in SKBR3 cells (Figure 4B). Consistently, phenformin induced similar changes in the expression of the EMT markers in BT474 cells, another ErbB2-overexperssing human breast cancer cell line (Supplementary Figure 3A). Phenformin also dose-dependently upregulated mRNA levels of *CDH1* with concomitant downregulation of *VIM*, *SNAI1*, and *SNAI2*, indicating that phenformin hinders EMT at the transcriptional level in breast cancer cells (Figure 4C). Taken together, these data suggest that the EMT regulatory network is sensitive to phenformin treatment.

### Phenformin inhibits EMT markers in syngeneic tumor grafts in vivo

**Figure 5:**
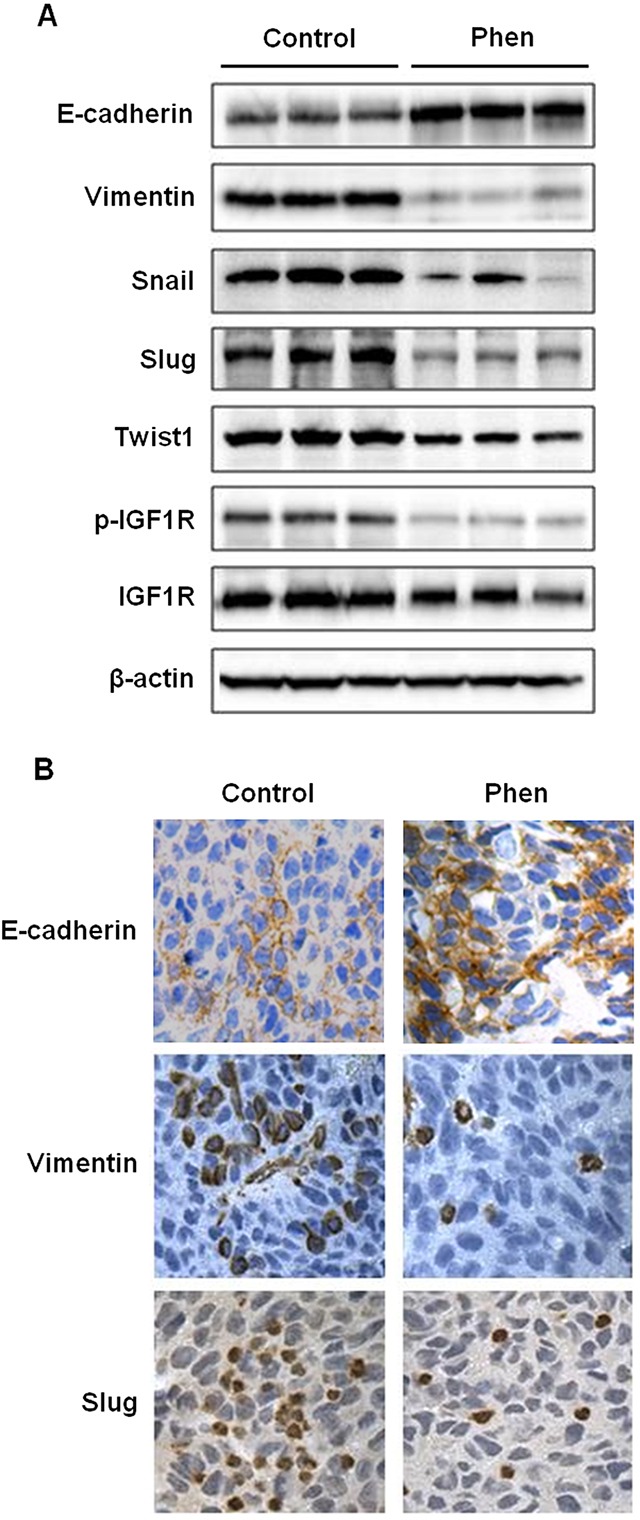
Phenformin inhibits EMT in ErbB2-overexpressing syngeneic tumor graft cells *in vivo* As previously described, syngeneic tumors from MMTV-ErbB2 transgenic mice treated with phenformin (0 or 30 mg/kg/day) for 20 days were collected and prepared for Western blot analysis and tissues were also paraffin-embedded for immunohistochemical analysis. **(A)** Western blot detection of EMT markers is shown for 3 representative samples from control and phenformin-treated mice. **(B)** Representative images from immunohistochemical analysis of E-cadherin, vimentin, and Slug in tissues from tumor grafts are shown at 400× magnification with brown staining indicating protein expression.

As an extension of our *in vitro* results, we studied the effects of phenformin on EMT in samples from our *in vivo* syngeneic tumor graft model. We analyzed the tumor tissues from mice treated with saline (control) or phenformin (30 mg/kg/day) for 20 days. Results from Western blotting revealed that the protein levels of E-cadherin were upregulated, while the protein levels of vimentin, Snail, Slug, and Twist1 were downregulated in tumor tissues from phenformin-treated mice (Figure 5A). These observations were verified by immunohistochemical staining, which also showed similar phenformin-induced increases in E-cadherin and decreases in vimentin and Slug protein expression as compared to the control mice (Figure 5B). These results demonstrate that phenformin-mediated tumor inhibition *in vivo* was associated with the modulation of the EMT process.

### Phenformin activates AMPK signaling and inhibits ErbB2 and IGF1R signaling pathways

**Figure 6:**
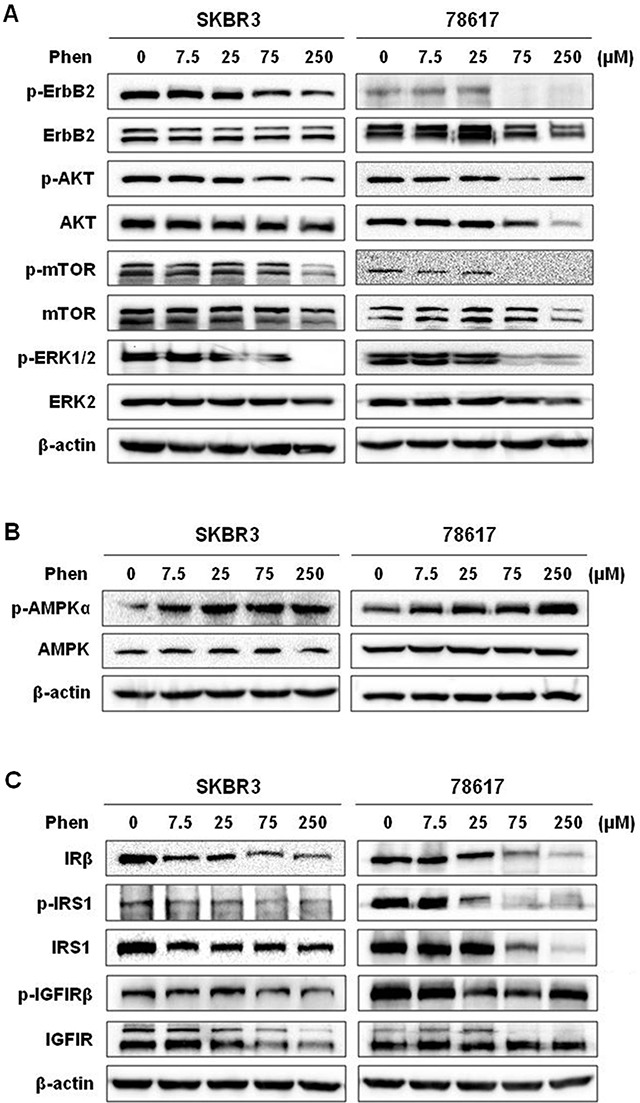
Phenformin activates AMPK signaling and inhibits ErbB2 and IGF1R signaling pathways Western blot analysis of ErbB2 activation and signaling (**A**), AMPK activation (**B**), and IRS/IGF1R activation and signaling (**C**) in SKBR3 and 78617 cells treated with phenformin (0, 7.5, 25, 75, or 250 μM) for 72 hours.

To understand the underlying mechanisms of phenformin-induced inhibition of tumor growth and EMT, we first examined the effects of phenformin on the ErbB2 signaling pathway, which is the driving force behind the oncogenicity of these tumors. We previously reported that metformin, a biguanide analog of phenformin, inhibits RTK signaling in breast cancer *in vitro* and *in vivo* [32]. Similarly, we observed that ErbB2-mediated signaling pathways were dose-dependently inhibited by phenformin, as indicated by decreased phosphorylation/activation of ErbB2, AKT, mTOR, and ERK (Figure 6A). In addition to the inhibition of the kinase activity in this pathway, there was a decrease in basal levels of certain markers after phenformin exposure, especially in 78617 cells, suggesting potential cell line-specific regulation of protein expression. Altogether, the downregulation of ErbB2 signaling is consistent with phenformin-induced growth inhibition and cell cycle arrest.

It has been reported that the activation of AMPK and consequential inhibition of mTOR activate metformin- and phenformin-induced cellular responses [33, 34]. Metformin- and phenformin-mediated inhibition of IR and IR substrate (IRS) leads to the suppression of IGF/IGF1R signaling [8, 9]. We therefore analyzed these markers in phenformin-treated ErbB2-overexpressing breast cancer cells. We observed here that low to high concentrations of phenformin (7.5 – 250 μM) induced robust phosphorylation of AMPK, while total AMPK levels remained unchanged after phenformin treatments in SKBR3 and 78618 cell lines (Figure 6B). Results also showed that phenformin substantially downregulated IRβ levels as well as the activation and expression of its substrate, IRS1 (Figure 6C). Moreover, phenformin decreased both the phosphorylation and protein expression of IGF1R in SKBR3 and 78617 cell lines (Figure 6C). These results imply that phenformin effectively blocked IR/IGF1R signal transduction.

### Phenformin inhibits IGF1-induced cell proliferation, RTK signaling, and EMT in ErbB2-overexpressing breast cancer cells

**Figure 7:**
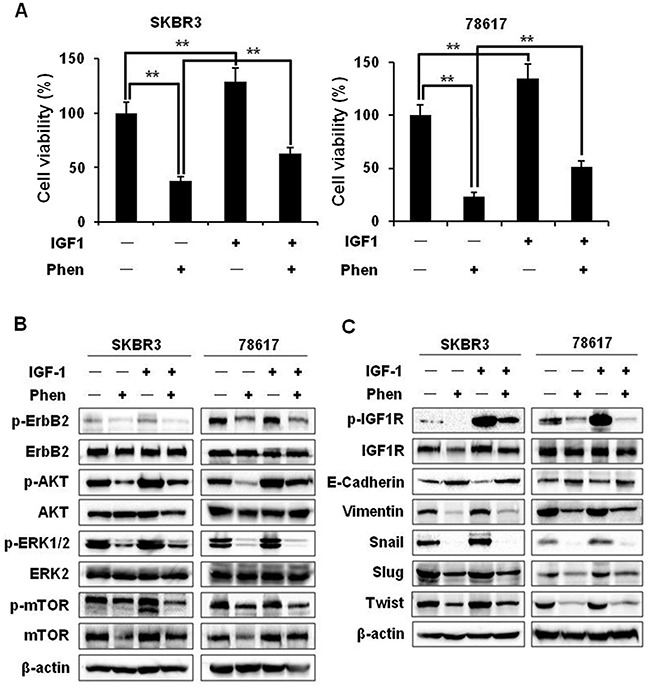
Phenformin inhibits IGF1-induced cell proliferation, RTK signaling, and EMT in ErbB2-overexpressing breast cancer cells **(A)** SKBR3 and 78617 cells were treated with IGF1 (100 ng/mL) and/or phenformin (150 μM) for 5 days. An MTT assay was performed to determine cell proliferation. Data are presented as the mean ± S.E. (** p<0.01). **(B)** SKBR3 and 78617 cells were treated with IGF1 (100 ng/mL) and/or phenformin (150 μM) for 24 hours and analyzed by Western blotting to detect the expression/activation of ErbB2, AKT, ERK, and mTOR. **(C)** SKBR3 and 78617 cells were treated with IGF1 (100 ng/mL) and/or phenformin (150 μM) for 48 hours and analyzed by Western blotting to detect the expression/activation of IGF1R and the expression of EMT markers.

To further explore the role of IGF1R signaling pathway in the anti-cancer activity and EMT modulation of phenformin, we examined the specific effects of phenformin on IGF1-induced proliferation, RTK signaling, and the alteration of EMT markers in SKBR3 and 78617 cells. MTT assay showed that IGF1 (100 ng/mL) promotes cell growth as compared to unstimulated control cells. However, in the presence of phenformin (150 μM), IGF1-stimulated growth was significantly inhibited, implying the specific impact of phenformin on IGF1R-mediated signaling (Figure 7A). We also found that phenformin inhibited IGF1-induced activation/phosphorylation of ErbB2, AKT, ERK, and mTOR (Figure 7B, Supplementary Figure 4), indicating the role of phenformin-IGF1R interactions in the modulation of RTK signaling. Importantly, phenformin significantly suppressed IGF1-induced IGF1R activation. It also reverted IGF1-induced downregulation of E-cadherin and upregulation of vimentin, Snail, Slug, and Twist (Figure 7C). To note, phenformin suppressed general IGF1-induced RTK activation and EMT marker expression in BT474 breast cancer cells as well (Supplementary Figure 3B-3C). These findings suggest that the suppression of IGF1R signaling is a critical step associated with phenformin-induced growth inhibition and EMT modulation in ErbB2-overexpressing breast cancer cells.

## DISCUSSION

In this study, we showed that phenformin stimulates cytotoxic and cytostatic effects in ErbB2-overexpressing breast cancer cells (Figure 1), which corroborates previous reports in other breast cancer cell lines [16, 34, 35]. From our initial data, 25 and 75 μM phenformin were approximately the IC_50_ values for 78617 and SKBR3 cells, respectively, and exhibited significant dose-dependent effects on the proliferative, apoptotic, and cell cycle endpoints. We also showed that phenformin inhibited cancer cell migration, invasion, and EMT markers (Figures 3, 4), which are linked to aggressive breast cancer phenotypes and metastatic potential. These effects were associated with the activation of AMPK and the blockage of RTK signaling (Figure 6). We further demonstrated that phenformin attenuated IGF1-stimulated cell proliferation, RTK signaling, and EMT *in vitro* (Figure 7). Consistently, data from our syngeneic model demonstrated that 30 mg/kg/day of phenformin, which was chosen based on literature that reported no toxic effects [36, 37], has potent inhibitory effects on tumor growth and EMT markers *in vivo* (Figures 2, 5).

In contrast to the extensive studies on metformin-associated anti-cancer activities, studies on phenformin-mediated anti-cancer effects are limited. Among the available reports, it was shown that phenformin can inhibit mammary tumorigenesis in carcinogen-induced breast cancer models in rats [15, 38]. Data from cell line models showed that phenformin triggered growth inhibition and apoptosis in various cancer cells as well [16, 34, 35]. For example, El-Masry *et al*. (2012) and Liu *et al*. (2015) both used multiple breast cancer cell lines with diverse genetic backgrounds (i.e. varying p53 and estrogen receptor statuses) to reveal the phenotypic effects of phenformin on different subtypes of breast cancer [16, 34]. We advanced these studies by particular investigation of phenformin-mediated responses in ErbB2-overexpressing breast cancer cells with additional mechanistic insight. The doses used in our *in vitro* and *in vivo* studies were also substantially lower than previous reports that tested doses ranging from 2.347 – 4 mM in breast cancer cell lines and up to 300 mg/kg/day in mouse models of breast cancer [16, 34, 39, 40]. In the context of previous reports, our data provide fundamental support of phenformin as a candidate for ErbB2^+^ breast cancer therapy.

Notably, epidermal growth factor (EGF), a ligand for EGFR-ErbB2 heterodimers, is involved in E-cadherin degradation, contributing to the conversion of epithelial cells to mesenchymal cells through EMT [17]. Upon the initiation of the EMT process by TGF-β and other factors, Snail and Slug, direct suppressors of E-cadherin, are activated along with downstream EMT markers (i.e. vimentin) [17]. Consequently, E-cadherin degradation or loss of function disrupts cell-cell adhesion, resulting in phenotypic cellular changes that are conducive for cell motility, which facilitates cell migration/invasion and promotes tumor metastasis [17, 19]. Our data from ErbB2-overexpressing breast cancer cells and syngeneic tumor grafts corroborate that phenformin can block EMT, as evidenced by the upregulation of E-cadherin and concurrent downregulation of EMT markers (Figures 4, 5). Our results suggest that the EMT process is a sensitive target of phenformin-induced anti-cancer activity. Furthermore, our previous report has demonstrated that cancer stem cells (CSCs), which are largely influenced by EMT [41], are selectively inhibited by metformin [32]. Intriguingly, phenformin may have similar CSC-targeting potential as a result of EMT inhibition. Nevertheless, our future phenformin studies will investigate the potential association between EMT modulation and CSC-targeted inhibition.

The constitutive activation of AMPK and inactivation of mTOR signaling after phenformin treatment was concurrent with the inhibition of RTK signaling in our study. While our data reiterate the link between AMPK and RTK signaling in phenformin-induced cellular activities, the fundamental mechanisms remain unclear. To shed light on these underlying mechanisms, we demonstrated that phenformin potently inhibits RTK signaling (Figure 6). As these pathways are critical for the survival of ErbB2-overexpressing breast cancer cells, our results not only explain the general inhibitory actions of phenformin, but also underscore the value of phenformin in ErbB2^+^ breast cancer treatments. Since AKT, ERK, and mTOR activation is downstream of several RTKs, including ErbB2 and IGF1R, it is interesting to elucidate the connection between phenformin treatment and RTK signaling inhibition [42]. We found that phenformin inhibited the activation of both ErbB2 and IGF1R (Figure 6). Previously, it was reported that ErbB2 and IGF1R can heterotrimerize with ErbB3 to enhance receptor activation and downstream signaling responses in ErbB2-overexpressing breast cancer cells [43]. It was also shown that IGF1R is downstream of IR and IRS in metformin-induced signaling regulation [44, 45]. As such, IGF1R signaling is perhaps suppressed by phenformin due to decreased insulin levels, which has been identified as a systemic effect of metformin and phenformin. Additionally, AMPK activation can also inhibit intracellular IRS1/2 activation/signaling, which includes the PI3K/AKT pathway [46]. We therefore reasoned that the inactivation of ErbB2, downstream AKT, ERK, and mTOR, and the subsequent inhibition of EMT and other pro-cancerous phenotypes, were secondary to phenformin-mediated suppression of IGF1R through IR and IRS downregulation. Moreover, modulation of IGF1R was reported to regulate EMT, particularly in breast cancer, and self-renewal of CSCs [23, 42, 47]. This was supported by our data showing that phenformin inhibited overall IGF1-stimulated cell proliferation, RTK signaling, and EMT (Figure 7). Since phenformin is not a specific IGF1 inhibitor, partial inhibition of some IGF1-induced RTK and EMT markers (e.g. p-AKT in 78617 cells and Snail in BT474 cells) is to be expected and may demonstrate potential cell line-specific responses. It should be noted that phenformin inhibits cancer cell growth and EMT in concert with the modulation of other pathways, which contributes to the anti-tumor effects of phenformin seen in breast cancer models. Collectively, our work builds the foundation for the multifaceted anti-cancer mechanisms of phenformin, with particular association between EMT modulation and RTK signaling.

Importantly, elevated IGF1R expression is prevalent in women who are at risk for developing breast cancer subtypes with distinctive aggressive phenotypes. In particular, IGF1R activation/overexpression, which appears in approximately 50% of all breast cancer cases and up to 65% of ErbB2^+^ breast cancers, is correlated with decreased survival in patients with ErbB2^+^ breast cancer—presumably because of the presence and activation of the IGF1R pathway for alternative downstream signaling [47–49]. Considering that IGF1R deregulation is not limited to ErbB2^+^ breast cancer, our data widen the scope of breast cancer subtypes that may respond to phenformin treatment to include ErbB2-negative breast cancer with IGF1R deregulation, yet further investigation is necessary.

Early clinical trials testing phenformin and buformin were focused on treating patients with diabetes. Unfortunately, these trials were terminated due to safety concerns associated with lactic acidosis before the potential anti-cancer properties of biguanides were fully realized. As such, the investigation of the anti-cancer benefits and underlying mechanisms, as well as optimization of treatment doses to reduce toxicity and develop an acceptable safety profile, remain incomplete for phenformin. Several potential dosing strategies need to be explored to fully exploit the anti-cancer benefits of phenformin. First of all, based on preclinical studies, phenformin has demonstrated more potent anti-cancer effects at lower doses than metformin. Therefore, the lowest effective dose of phenformin as a single anti-cancer agent, as well as the safety profiles for the lower doses, needs to be determined. In addition to lowering the dose of phenformin to reduce the occurrence of toxic side effects, alternative strategies have been explored using phenformin in combination with other agents. For instance, an initial report has shown that phenformin in combination with 2-deoxyglucose can act synergistically to suppress colon cancer cell growth and reduce acidification *in vitro* [50]. Finally, low doses of phenformin should be tested with other anti-cancer therapeutic combinations to determine any synergistic effects. It is important to note that a clinical trial is ongoing/recruiting to test the potential therapeutic use of phenformin in combination with dabrafenib + trametinib in patients with metastatic melanoma (ClinicalTrials.gov NCT03026517).

Incidentally, more preclinical and clinical testing to determine safe and effective doses of phenformin may expand the clinical application of phenformin as a breast cancer preventative, combination therapy, or adjuvant therapy. In this regard, implementation of phenformin as a cancer preventative in women who are at risk for developing ErbB2^+^ breast cancer would provide substantial clinical benefits. Moreover, despite multiple ErbB2-targeted therapeutics currently approved and in clinical trials, ErbB2^+^ breast cancers are associated with major clinical challenges due to the development of drug resistance. As such, IGF1R-ErbB2 crosstalk is strongly linked to ErbB2-targeted drug resistance. Indeed, trastuzumab resistance is a common problem in ErbB2^+^ breast cancers because tumor cells can circumvent ErbB2 inhibition by activating IGF1R [47, 51]. Our data denoting that phenformin inhibits IGF1R and ErbB2 additionally fortify phenformin as a potential therapeutic in drug-resistant ErbB2^+^ tumors. Furthermore, we used phenformin as a single agent, but, in the context of other reports, phenformin also shows synergistic effects with other anti-cancer drugs, like combinational therapy with vemurafenib, a BRAF inhibitor, in melanoma cells [52, 53].

Overall, our study demonstrates that phenformin has potent, multifaceted anti-tumor effects, such as suppression of growth, cell cycle, migration, invasion, and EMT. The phenformin-induced inhibition of these pro-cancerous cellular responses was shown to be mediated through inactivation of IGF1R and ErbB2 signaling. Our data provide supportive evidence of the anti-cancer properties of phenformin and underscore the potential of phenformin and other biguanide drugs as effective cancer preventative and therapeutic strategies. Additional mechanistic insight and testing relevant to different clinical settings will facilitate the clinical implementation of phenformin as a cancer therapeutic agent.

## MATERIALS AND METHODS

### Reagents and antibodies

Phenformin hydrochloride was purchased from Sigma-Aldrich (St. Louis, MO). IGF1 was purchased from R&D Systems (Minneapolis, MN). Antibodies against AMPK, p-AMPKα (T172), ErbB2, p-ErbB2 (Y877), AKT, p-AKT (S473), mTOR, p-mTOR (Ser2448), p-ERK1/2 (T202/Y204), IGF1R, p-IGF1Rβ (T1135/1136), IRβ, IRS1, p-IRS1 (Ser307), E-cadherin, vimentin, Slug, and Snail were purchased from Cell Signaling Technology (Danvers, MA). Antibodies against ERK2 and β-actin were purchased from Santa Cruz Biotechnology (Santa Cruz, CA). Twist1 antibody was obtained from Sigma-Aldrich.

### Cell culture and treatments

The human breast cancer cell line SKBR3 was purchased from American Type Culture Collection (ATCC; Manassas, VA). The 78617 cell line is a mammary tumor cell line derived from MMTV-ErbB2 transgenic mice with spontaneous tumors expressing high levels of ErbB2, as previously described [31]. The cells were maintained in DMEM/F-12 medium (Invitrogen; Grand Island, NY) supplemented with 10% fetal bovine serum (FBS; Invitrogen), penicillin (100 U/mL), and streptomycin (100 μg/mL) in a humidified atmosphere of 5% CO_2_ at 37°C. For IGF1 treatments, SKBR3 and 78617 cells were starved in serum-free medium (SFM) for 24 hours prior to treatment with IGF1 (100 ng/mL). After 1 hour of IGF1 exposure, phenformin (150 μM) was then added to the medium. Other cell treatments are indicated in individual experiments.

### Animals and treatments

For the syngeneic tumor grafting experiment, 78617 cells were cultured in complete DMEM/F12 medium and harvested in the logarithmic phase of growth. After adjusting cell number based on viability, 1×10^6^ viable cells were subcutaneously injected into the flank of female 8-week-old MMTV-ErbB2 transgenic mice (*n*=8 for each of the control and phenformin-treated groups). Phenformin (30 mg/kg/day) was administered via an intraperitoneal injection into mice from the treatment group for 20 days. Tumor volumes were measured every other day, beginning on the 8th day after the initial intraperitoneal injection until the 20th day. The tumor volume was calculated as: Tumor volume = longest diameter × shortest diameter^2^× 0.5. Syngeneic tumor grafts were removed and processed for further analysis. All procedures involving mice were performed with the approval of the University's Institutional Animal Care and Use Committee and conducted in accordance with the NIH Guide for the Care and Use of Laboratory Animals.

### Cell proliferation assay

The cells were seeded (1×10^3^ cells/well) in 96-well plates and then treated with phenformin the next day, at indicated concentrations, for 5 days [35]. After 5 days, cell viability was assessed using a CellTiter 96 Non-Radioactive Cell Proliferation kit (Promega; Madison, WI). The medium was replaced with 50 μl of 3-(4,5-dimethythiazol-2-yl)-2,5-diphenyltetrazolium bromide (MTT; 2.5 mg/mL) in SFM, followed by a 4 hour incubation at 37°C. The MTT solution was then replaced with 50 μl of DMSO, followed by incubation on a shaker to dissolve the formazan crystals. The absorption was measured with a microplate reader at 540 nm. The cell viability of each group, based on 8 parallel samples, was calculated relative to the controls, which were normalized to 100% survival.

### Clonogenic assay

Cells in the logarithmic phase of growth were collected for inoculation and were seeded (1×10^3^ cells/well) in 6-well plates. Then the cells were treated with phenformin at indicated concentrations for 2 weeks. The medium was changed once on the 3^rd^ day during the 2 week treatment. Following the phenformin treatment, the culture dishes were washed twice with phosphate buffered saline (PBS), fixed with acetone/methanol (1:1) for 5 minutes, and incubated with 0.5% crystal violet for 20 minutes at room temperature. After aspirating the excess stain, the plates were washed with deionized water and air-dried. The colonies were imaged with a FluorChemE imager (Cell Biosciences; Santa Clara, CA) and analyzed with AlphaView software.

### Apoptosis assay

Apoptosis was determined using an Annexin V-FITC Apoptosis Detection kit from BD Biosciences (San Jose, CA) according to the manufacturer's instructions. Briefly, the cells were cultured in phenformin for 48 hours, and then were collected by trypsinization. After washing with PBS, the cells were stained with Annexin V-FITC and propidium iodide (PI) and then detected by flow cytometry. The percentages of apoptotic cells in each group, including cells in the early (Annexin V^+^/PI^−^) and late (Annexin V^+^/PI^+^) stages of apoptosis, were quantified based on three independent experiments.

### Cell cycle analysis

The cells were treated with phenformin at different concentrations for 24 hours, followed by trypsinization and collection. The single cell suspension was fixed with 70% ethanol (added drop-wise) and stored overnight at −20°C. The fixed cells were then washed twice with ice-cold PBS and stained with PI (33 μg/mL with 0.1% Triton X-100) in the presence of 500 μg/mL RNase A in the dark for 1 hour at room temperature. DNA content was analyzed on a guava EasyCyte 8 flow cytometer (EMD Millipore; Billerica, MA). The derived data were analyzed with ModFit LT Software to estimate the percentages of cells at G0/G1, S, and G2/M phases.

### Wound healing assay

Cells were seeded in 6-well plates and cultured to 90-100% confluence in SFM. A single wound was then made in the monolayer of cells with a pipette tip. After washing twice with PBS to remove debris, the cells were cultured in the absence or presence of phenformin at various concentrations for 24 hours. Images of the same area of the wound were taken at 100× magnification at 0 hours and 24 hours after the wound was made. The percentage of migration was calculated as the difference between the wound width at 0 hours and 24 hours. The wound width at 0 hours was normalized to 100%. Each experiment was performed in triplicate.

### Invasion assay

*In vitro* cell invasion activity was detected with a Biocoat Matrigel Invasion Chamber kit from BD Biosciences, according to the manufacturer's instructions. Briefly, 2.5×10^4^ cells in SFM were added into the upper chamber of a matrigel pre-coated insert. The lower chamber was filled with 750 μL of DMEM/10% FBS medium-treated with phenformin at indicated concentrations. After 24 hours, the cells remaining on the upper surface of the membrane were removed with a cotton swab. The cells that had invaded through the membrane were stained with methanol and 0.2% crystal violet, followed by image capture using a Nikon SMZ 745T microscope at 40× magnification. For quantification of invading cells, cells in five randomly selected microscopic fields were counted. The data were based on three independent experiments.

### Immunofluorescence

On glass coverslips, cells were seeded (2×10^4^ cells/well) in 24-well plates in SFM overnight. Next, the cells were treated with or without phenformin (75 μM) for 24 hours, followed by 4% paraformaldehyde fixation for 15 minutes and PBS washes at room temperature. For morphological analysis, cells were permeabilized with 0.5% Triton X-100 for 15 minutes, blocked with 1% bovine serum albumin (BSA; Sigma-Aldrich) in PBS for 30 minutes at room temperature, and then incubated with primary antibodies of anti-E-cadherin and vimentin (diluted 1:100 in 1% BSA) at 4°C overnight. The next day, Alexa Fluor 488-conjugated goat monoclonal anti-rabbit IgG secondary antibody (Molecular Probes; Eugene, OR) was added in blocking solution in the dark for 1 hour at room temperature. Cell nuclei were stained with 4′,6-diamidino-2-phenylindole (DAPI) for 15 minutes and then the cells were mounted with Vectashield mounting gel media (Vector Laboratories; Burlingame, CA). The cells were viewed and imaged using a LSM880 (Zeiss) confocal microscope with AxioObserver.

### Western blot analysis

Cells were collected for lysate preparation as previously described [54]. Protein concentrations were determined with the BCA Protein Assay kit from Thermo Scientific Pierce (Rockford, IL). Protein (50 μg) from each sample was separated with SDS-PAGE and transferred to a nitrocellulose membrane. The membrane was blocked with 5% milk in TBST buffer, followed by incubation with a primary antibody at 4°C overnight. After washing with TBST, the membrane was incubated with the corresponding horseradish peroxidase-labeled secondary antibody for 2 hours at room temperature. After washing with TBST again, proteins were detected using enhanced chemiluminescence (ECL) reagents (Thermo Scientific Pierce). The images were captured with a FluorChemE imager.

### RNA isolation and quantitative polymerase chain reaction (qPCR)

Total RNA was extracted from cells using TRIzol reagent (Invitrogen) and reverse transcription was performed using an iScript cDNA Synthesis Kit (Bio-Rad). qPCR was carried out with the Bio-Rad CFX96 Touch Real-Time PCR Detection System using GeneCopoeia All-in-One qPCR Mix (GeneCopoeia; Rockville, MD) according to the manufacturer's instructions. The PCR conditions were as follows: 94°C for 2 minutes, 40 cycles of 94°C for 30 seconds, 56°C for 30 seconds, and 72°C for 30 seconds. The primers sequences for each gene analyzed are listed in Supplementary Table 1. Glyceraldehyde-3-phosphate dehydrogenase (*GAPDH*) and β-actin (*actb*) were used as an internal controls for human and mouse samples, respectively. The relative expression of each gene was calculated and normalized, using the 2^−ΔΔCt^ method, as compared to the expression in control samples.

### Immunohistochemical staining

Tumor specimens were derived from formalin-fixed, paraffin-embedded tissue samples obtained from mice with syngeneic tumor cell grafts. Immunohistochemistry was performed as previously reported [55]. Following antigen retrieval and blocking, tumor tissues were incubated in E-cadherin (diluted 1:100), vimentin (diluted 1:100), and Slug (diluted 1:50) antibodies overnight at 4°C. The slides were then incubated with biotinylated anti-rabbit IgG secondary antibody (Vectastain Elite ABC Kit, Vector Laboratories) for 30 minutes at room temperature, followed by incubation in Vectastain Elite ABC Reagent for 30 minutes at room temperature. For all slides, a diaminobenzidine detection kit (Vector Laboratories) was used according to the manufacturer's instructions. The sections were then counterstained with Meyer's hematoxylin, dehydrated, and mounted. Slides were observed and imaged using a Nikon ECLIPSE E100 microscope.

### Statistical analysis

Data from each experiment were obtained from at least three independent experiments. The significant differences between the control and individual experimental groups were determined using a Student's t-test. The Wilcoxon rank sum test was used to determine significant differences between control and phenformin-treated mice. Statistical analyses were performed using STATA 11 (StataCorp LP; College Station, TX). Differences with p<0.05 were considered statistically significant.

## SUPPLEMENTARY FIGURES AND TABLE



## Abbreviations

MTT: 3-(4,5-dimethythiazol-2-yl)-2,5-diphenyltetrazolium bromide
DAPI: 4′,6-diamidino-2-phenylindole
ATCC: American Type Culture Collection
AMPK: AMP-activated protein kinase
BSA: bovine serum albumin
CSCs: cancer stem cells
ECL: enhanced chemiluminescence
EGF: epidermal growth factor
EGFRs: epidermal growth factor receptors
EMT: epithelial-mesenchymal transition
ErbB2^+^: ErbB2-positive
FBS: fetal bovine serum
GAPDH: glyceraldehyde-3-phosphate dehydrogenase
IGF: insulin growth factor
IGFR: insulin-like growth factor receptor
IR: insulin receptor
IRS: insulin receptor substrate
mTOR: mammalian target of rapamycin
PBS: phosphate buffered saline
PI: propidium iodide
qPCR: quantitative polymerase chain reaction
RTK: receptor tyrosine kinase
SFM: serum-free medium
S.E.: standard error of the mean
TGF-β: tumor growth factor-β
