# Supplementary material for: Phenformin inhibits growth and epithelial-mesenchymal transition of ErbB2-overexpressing breast cancer cells through targeting the IGF1R pathway
Source: Oncotarget. 2017 Jul 22;8(36):60342–57. doi: 10.18632/oncotarget.19466 (PMC5601143; doi:10.18632/oncotarget.19466)
Supplement: Supplementary file 1 [file oncotarget-08-60342-s001.pdf]

Phenformin inhibits growth and epithelial-mesenchymal transition of ErbB2-overexpressing breast cancer cells through targeting the IGF1R pathway

SUPPLEMENTARY MATERIALS

Wound healing assay with mitomycin C

Cells were seeded in 6-well plates and cultured to 90-100% confluence in SFM. A single wound was then made in the monolayer of cells with a pipette tip. After washing twice with PBS to remove debris, the cells were pretreated with mitomycin C (10 µM) for 1 hour to inhibit cell proliferation before phenformin treatment (75 µM for 24 hours) (Supplementary Figure 2A). Images of the same area of the wound were taken at 100× magnification at 0 hours and 24 hours after the wound was made. The edges of the wound are marked by dashed lines to indicate the wound width at 0 hours and 24 hours for each treatment group.

Boyden chamber assay

The Boyden chamber migration assay (8 µm pore size) was performed according to the manufacturer's instructions (Coring). Briefly, 1×10^5 (SKBR3) and 5×10^4 (78617) cells were seeded in SFM treated with phenformin (0, 25, or 75 µM) in the upper chamber of the transwell inserts and the lower wells were filled with complete DMEM/10% FBS medium. After 24 hours of incubation, the cells remaining on the upper surface of the membrane were removed with a cotton swab, and the migrated cells on the lower surface of the membranes were stained with methanol and 0.2% crystal violet, followed by image capture using a Nikon SMZ 745T microscope at 40× magnification (Supplementary Figure 2C).

BT474 cell culture and treatment

The human breast cancer cell line BT474 was purchased from ATCC and cultured in DMEM/F-12 medium (Invitrogen) supplemented with 10% FBS (Invitrogen), penicillin (100 U/mL), and streptomycin (100 µg/mL) in a humidified atmosphere of 5% CO_2 at 37°C. For the dose-course study of phenformin on EMT markers, BT474 cells were treated with phenformin (0, 7.5, 25, 75, or 250 µM) for 72 hours, followed by Western blot analysis of the indicated markers. For IGF1 treatments, BT474 cells were starved in SFM for 24 hours prior to treatment with IGF1 (100 ng/mL). After 1 hour of IGF1 exposure, phenformin (150 µM) was then added to the medium for the indicated timepoints.

Densiometry

The optical densities (OD) of the indicated protein bands from 3 independent experiments were quantified using Quantity One software (Bio-Rad). The relative band densities were calculated as the ratio of the protein band OD relative to the β-actin (loading control) band OD from the same sample. The ratios for the untreated control samples were set to 1 (Supplementary Figure 4).

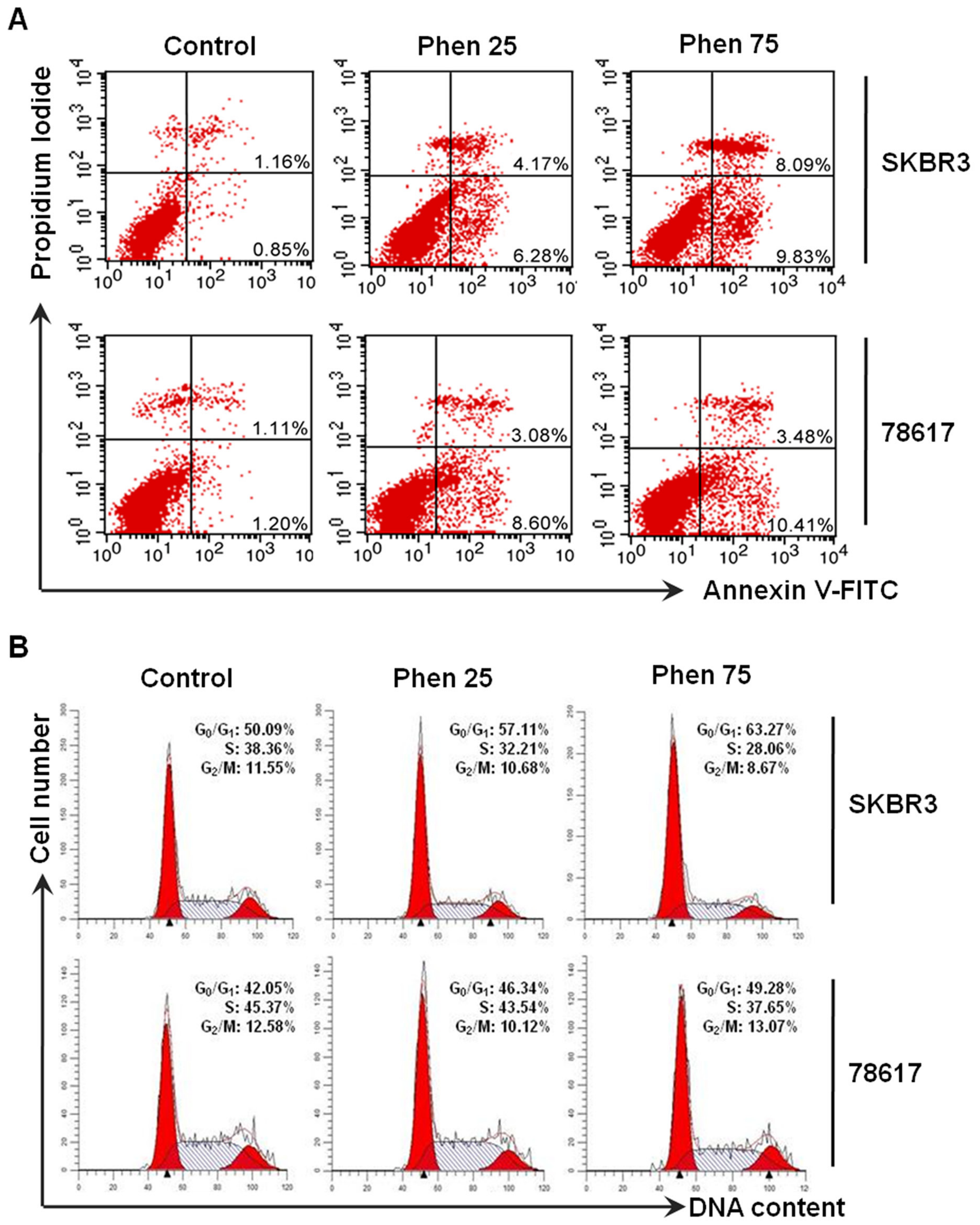

Supplementary Figure 1: Phenformin induces apoptosis and cell cycle arrest. Representative plots from apoptosis (**A**) and cell cycle (**B**) assays are shown.

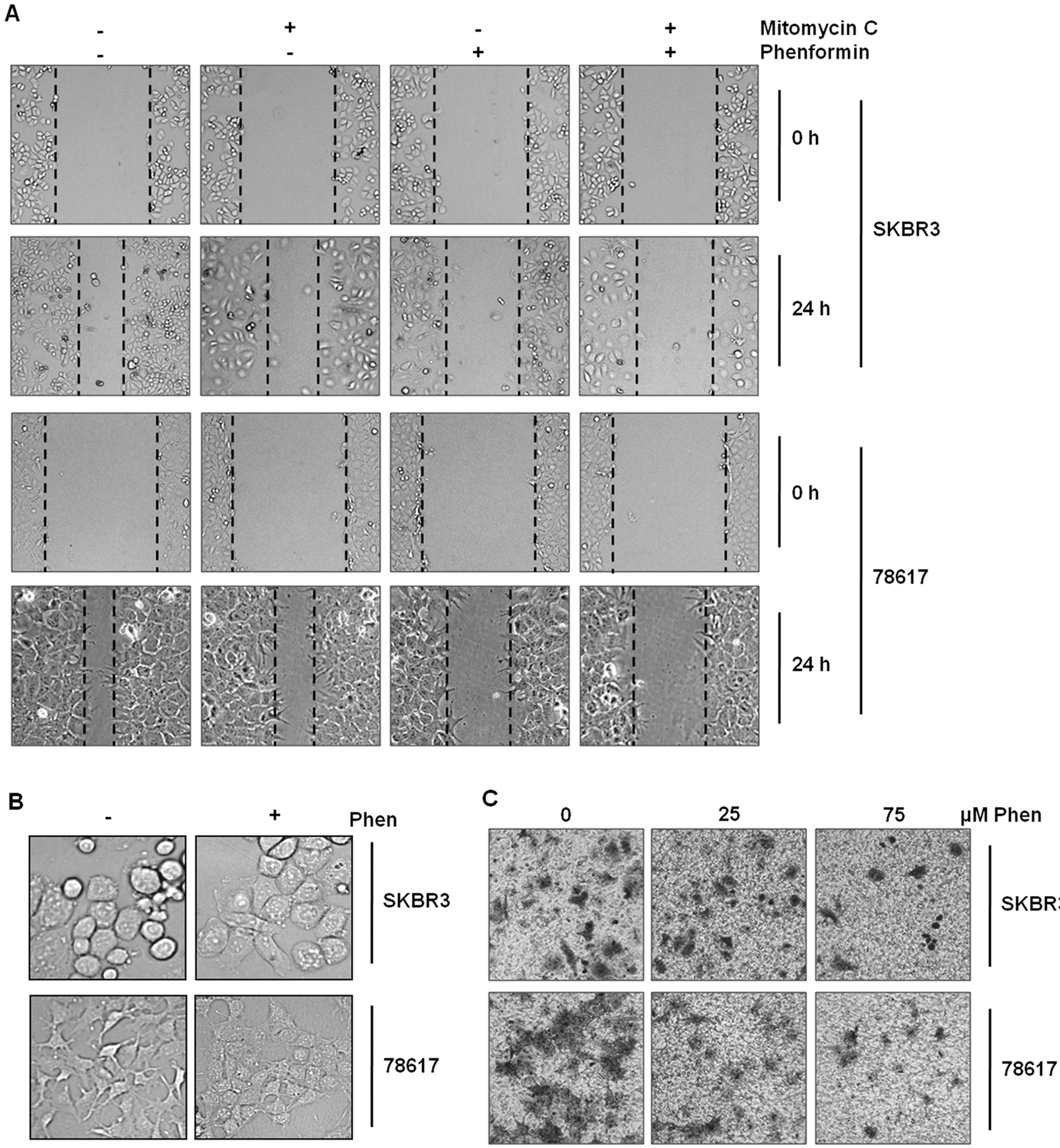

Supplementary Figure 2: Phenformin inhibits cell migration in the presence of mitomycin C and cell invasion in the absence of matrigel. **(A)** Wound healing assay of SKBR3 and 78617 cells pretreated with mitomycin C (10 µM for 1 hour), followed by phenformin treatment (75 µM for 24 hours) was performed as described in the *Supplementary Materials*. **(B)** Representative images depicting the morphology of SKBR3 and 78617 cells treated with phenformin (75 µM for 48 hours) are shown. **(C)** Boyden chamber assay of SKBR3 and 78617 was performed in SFM with phenformin treatments (0, 25, or 75 µM) for 24 hours. After 24 hours of incubation, the migrated cells were stained and imaged as detailed in the *Supplementary Materials*.

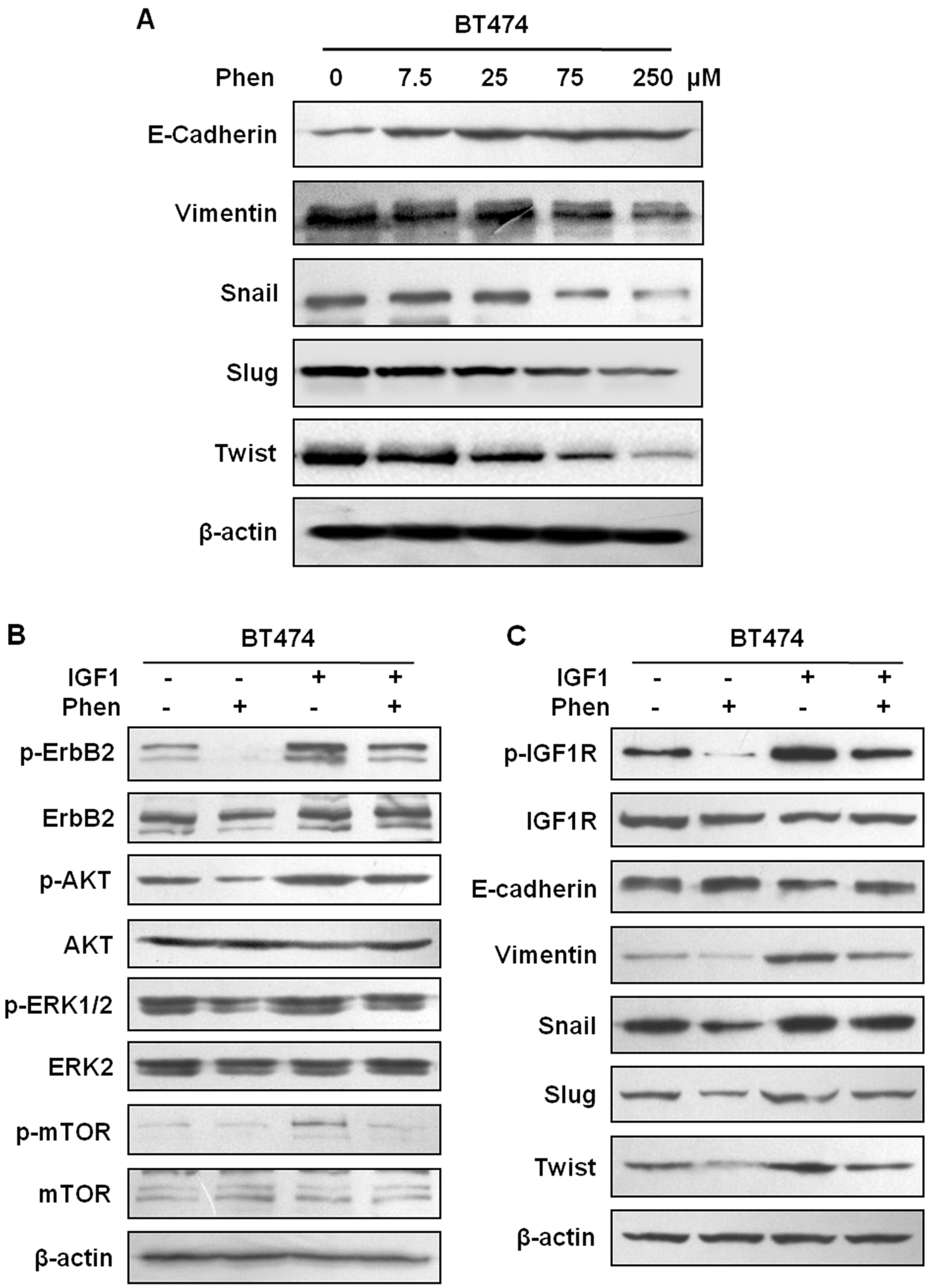

Supplementary Figure 3: Phenformin inhibits IGF1-induced RTK signaling and EMT markers in BT474 ErbB2-overexpressing human breast cancer cells *in vitro*. **(A)** Western blot analysis of EMT markers in BT474 cells treated with phenformin (0, 7.5, 25, 75, or 250 μM) for 72 hours is shown. **(B)** BT474 cells were treated with IGF1 (100 ng/ml) and/or phenformin (150 μM) for 24 hours and analyzed by Western blotting to detect the expression/activation of ErbB2, Akt, ERK, and mTOR. **(C)** BT474 cells were treated with IGF1 (100 ng/ml) and/or phenformin (150 μM) for 48 hours and analyzed by Western blotting to detect the activation of IGF1R and the expression of EMT markers.

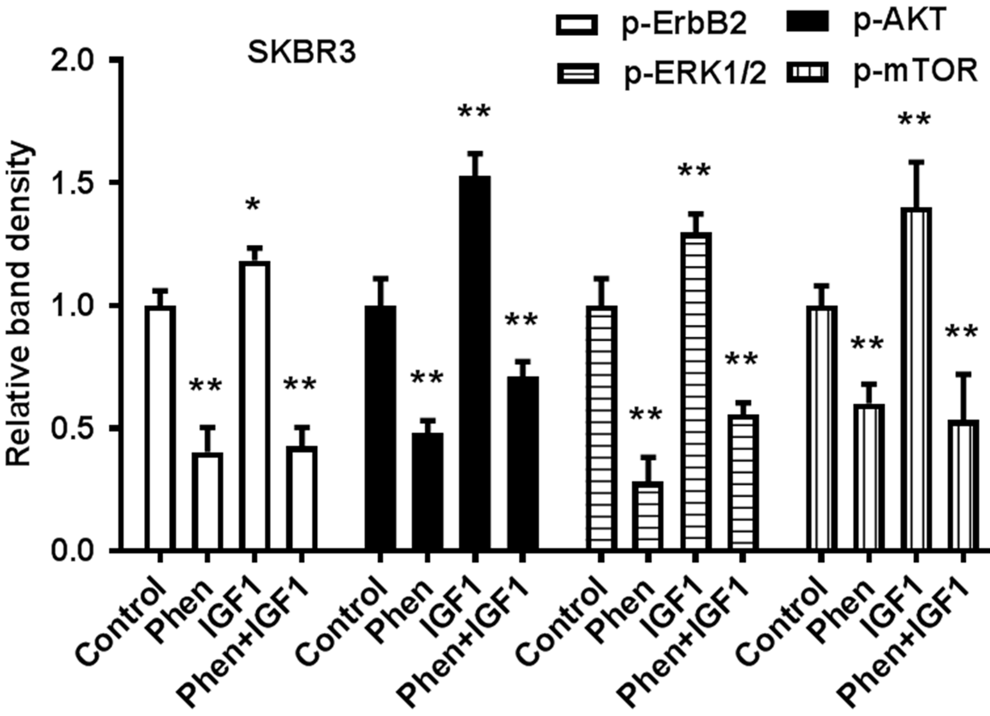

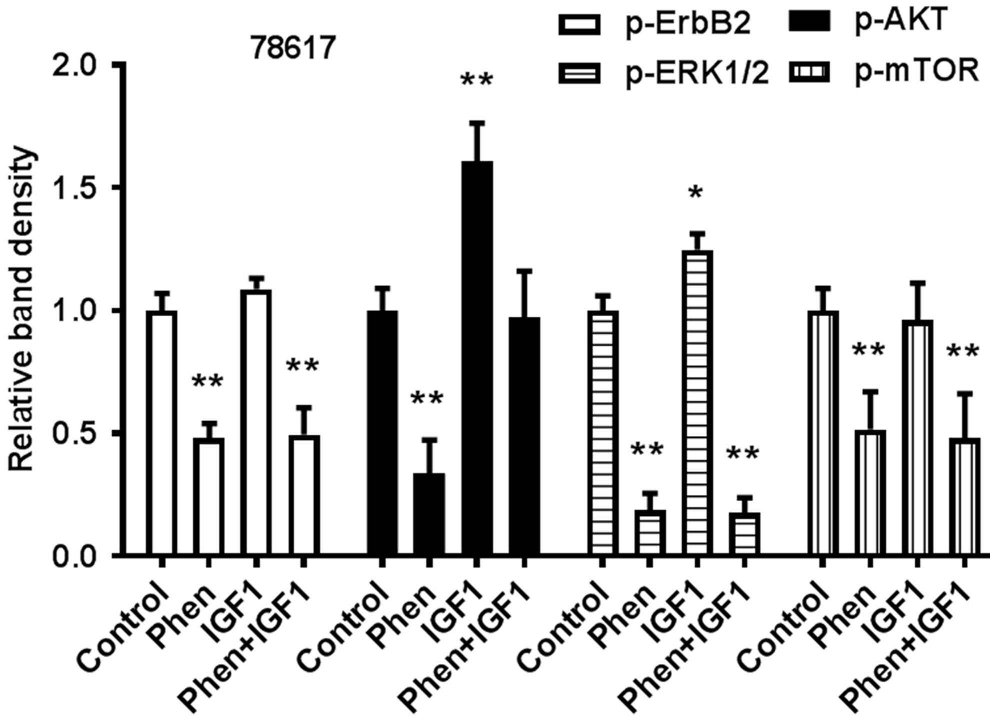

Supplementary Figure 4: Band density of phosphorylated proteins with IGF1 stimulation +/- phenformin treatment. The band density was quantified for phosphorylated ErbB2, AKT, ERK1/2, and mTOR as compared to the β-actin controls in SKBR3 and 78617 cells that were treated with IGF1 (100 ng/ml) and/or phenformin (150 µM) for 24 hours. Data are presented as the mean ± S.E. (* $p<0.05$, ** $p<0.01$ as compared to the corresponding controls).

Supplementary Table 1: Primer sequences used for qPCR

Protein name	Gene name	For human samples	For mouse samples
E-cadherin	CDH1	F: 5'-GACCGGTGCAATCTTCAAA-3'<br>R: 5'-TTGACGCCGAGAGCTACAC-3'	F: 5'-AAAAGAAGGCTGTCCTTGGC-3'<br>R: 5'-GAGGTCTACACCTTCCCGGT-3'
Vimentin	VIM	F: 5'-ATTCCACTTTGCGTTCAAGG-3'<br>R: 5'-CTTCAGAGAGAGGAAGCCGA-3'	F: 5'-TCCACTTTCCGTTCAAGGTC-3'<br>R: 5'-AGAGAGAGGAAGCCGAAAGC-3'
Snail	SNAI1	F: 5'-AGGTTGGAGCGGTCAGC-3'<br>R: 5'-CCTTCTCTAGGCCCTGGCT-3'	F: 5'-AGTGGGAGCAGGAGAATGG-3'<br>R: 5'-CTTGTGTCTGCACGACCTGT-3'
Slug	SNAI2	F: 5'-TGACCTGTCTGCAAATGCTC-3'<br>R: 5'-CAGACCCTGGTTGCTTCAA-3'	F: 5'-GATGTGCCCTCAGGTTTGAT-3'<br>R: 5'-GGCTGCTTCAAGGACACATT-3'
GAPDH	GAPDH	F: 5'-AATGAAGGGGTCATTGATGG-3'<br>R: 5'-AAGGTGAAGGTCGGAGTCAA-3'	
β-actin	actb		F: 5'-ATGGAGGGGAATACAGCCC-3'<br>R: 5'-TTCTTTGCAGCTCCTTCGTT-3'
